# Loss of palmitoylation impacts the interaction between the organic anion transporting polypeptide (OATP) 1B1 and OATP1B3

**DOI:** 10.1016/j.dmd.2026.100330

**Published:** 2026-05-16

**Authors:** Cecilia E. Villanueva, Whitney M. Nolte, Priscilla Flores-Ascencio, Bruno Hagenbuch

**Affiliations:** 1 Department of Pharmacology, Toxicology and Therapeutics, The University of Kansas Medical Center, Kansas City, Kansas; 2 Division of Clinical Pharmacology, Toxicology and Therapeutic Innovation, Children’s Mercy Hospital, Kansas City, Missouri

**Keywords:** Palmitoylation, OATP1B1, OATP1B3, Post-translational modification, Protein-protein interactions

## Abstract

The organic anion transporting polypeptide (OATP) 1B1 and OATP1B3 are hepatic transporters that mediate the uptake of numerous therapeutics like antibiotics and statins. Various post-translational modifications, eg, phosphorylation and glycosylation, have been shown to impact OATP1B1 and OATP1B3 function and localization. *S*-palmitoylation (also known as *S*-acylation or palmitoylation) is a reversible post-translational modification that adds palmitic acid (C16:0) to cysteine residues and can influence a protein’s function, localization, and protein-protein interactions. In this study, we investigated and characterized palmitoylation in OATP1B1 and OATP1B3. Using site-directed mutagenesis and acyl-resin–assisted capture assays, residue Cys24 was identified as the palmitoylation site for both these proteins. Uptake function and surface biotinylation experiments demonstrated that palmitoylation does not affect the individual function and surface expression of OATP1B1 and OATP1B3. Coexpression studies revealed a decrease in OATP1B1-OATP1B3 interactions when nonpalmitoylated OATP1B1 or nonpalmitoylated OATP1B3 are coexpressed with their wild-type counterparts, suggesting that *S*-palmitoylation regulates protein-protein interactions between OATP1B1 and OATP1B3.

**Significance Statement:**

This study identified and characterized palmitoylation of the organic anion transporting polypeptide (OATP) 1B1 and OATP1B3. Both OATP1B1 and OATP1B3 can be palmitoylated on the conserved Cys24 residue. Palmitoylation of these 2 transporters does not impact their individual function or surface expression. However, the interaction between these 2 proteins is affected when their palmitoylation state is altered.

## Graphical abstract

**Figure:**
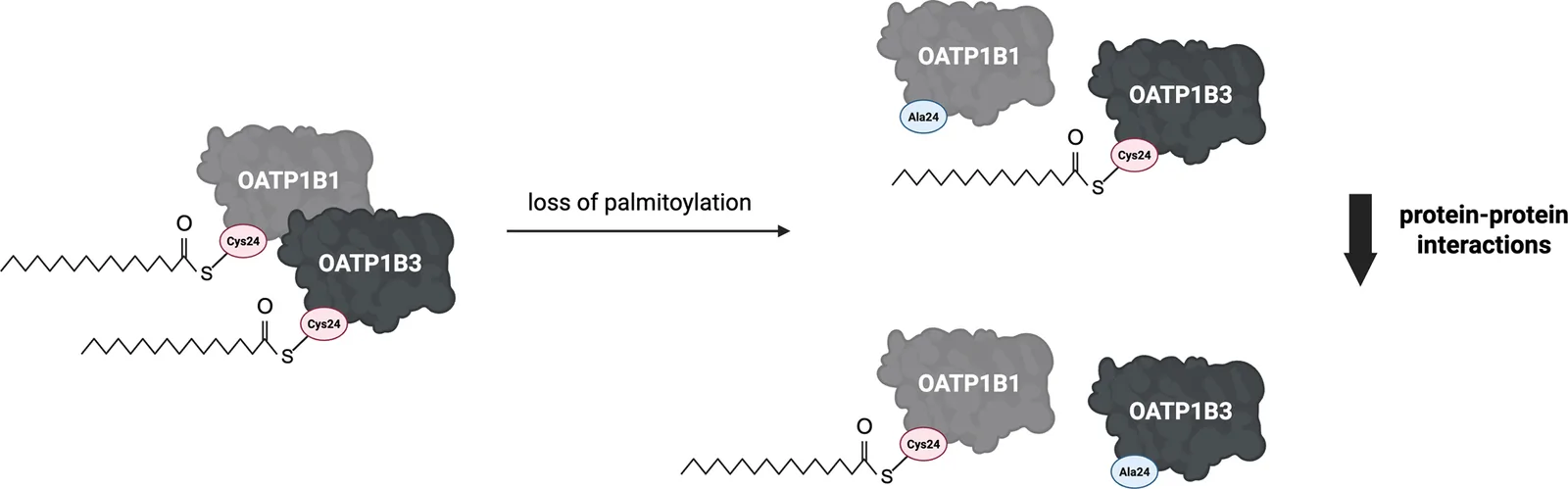

## 1 Introduction

Membrane transporters facilitate the transport of various endogenous and exogenous compounds throughout the body. Transporters play a vital role in drug disposition due to their ability to affect the bioavailability of drugs. The organic anion transporting polypeptide (OATP) 1B1 and OATP1B3 transporters share 80% amino acid sequence homology and are localized to the basolateral membrane of hepatocytes, where they mediate the uptake of numerous endobiotics and xenobiotics, including drugs like statins, chemotherapy agents, and antibiotics.^1^ OATP1B1 and OATP1B3 hetero-oligomerize, which can alter their function and expression.^2^^,^^3^ Due to the essential role OATP1B1 and OATP1B3 have on the bioavailability and pharmacokinetics of numerous therapeutic agents, the US Food and Drug Administration emphasizes that if hepatic metabolism or biliary secretion contributes to more than 25% of the potential drug’s clearance, interactions that may lead to OATP1B1 and OATP1B3 inhibition need to be investigated during new drug development.^4^^,^^5^ Therefore, due to the significant role that OATP1B1 and OATP1B3 play in drug disposition, a better understanding of how different modifications and mechanisms affect their function or localization is crucial.

Several post-translational modifications (PTMs) affect the function and localization of OATP1B1 and OATP1B3. For example, both are *N*-glycosylated.^6^, ^7^, ^8^ *N*-glycosylation is a PTM that results in the addition of oligosaccharides to asparagine residues via oligosaccharyl-transferase enzymes that can influence protein trafficking to the plasma membrane. Loss of *N*-glycosylation in OATP1B1 and OATP1B3 is linked to reduced protein plasma membrane localization,^6^^,^^9^^,^^10^ and is observed in disease states like metabolic dysfunction–associated steatotic liver disease.^11^ Phosphorylation is another PTM that regulates OATP1B1 and OATP1B3.^12^, ^13^, ^14^ OATP1B1 is phosphorylated on various tyrosine and serine residues. Tyrosine kinase inhibitors and protein kinase C inhibitors alter OATP1B1 function, suggesting that OATP1B1 phosphorylation is vital for its normal function and may also play a role in OATP1B1 internalization and recycling.^9^^,^^12^^,^^13^ Like OATP1B1, OATP1B3 is also phosphorylated by protein kinase C, which reduces its transport function.^6^^,^^14^

*S*-palmitoylation, also called *S*-acylation or palmitoylation, is a reversible PTM that adds palmitic acid (C16:0) to cysteine residues of proteins. Adding palmitic acid to a protein increases its hydrophobicity, which can impact its localization. This can facilitate protein docking into cellular membranes, particularly those enriched in cholesterol and sphingolipids, commonly referred to as lipid rafts.^15^, ^16^, ^17^ *S*-palmitoylation can also impact a protein's function and its interactions with other proteins. A vast number of proteins in the human body, including various transporters such as the human apical sodium-dependent bile acid transporter,^18^^,^^19^ the glucose transporter (GLUT) 4,^20^^,^^21^ and the sodium-calcium exchanger (NCX1),^22^ are *S*-palmitoylated.^23^

Previous studies from our lab suggest that both OATP1B1 and OATP1B3 are localized to positive lipid raft marker fractions of detergent-resistant membrane domains.^24^ Because proteins in lipid rafts are commonly lipidated, and their *S*-palmitoylation state impacts their membrane association, we wondered if this PTM may play a role in OATP1B1 and OATP1B3 localization, function, and protein interactions. Specifically, we hypothesized that OATP1B1 and OATP1B3 are palmitoylated and that this PTM impacts their function, surface expression, and their interactions with each other.

## 2 Materials and methods

### 2.1 Cell culture and transient transfection of transporters

Human embryonic kidney 293T/17 (HEK293) cells obtained from American Type Culture Collection (Manassas, VA) were grown and maintained at 37 °C with 5% CO_2_ in Dulbecco’s modified Eagle’s medium (American Type Culture Collection). The media was supplemented with 100 U/mL penicillin, 100 *μ*g/mL streptomycin (Thermo Fisher Scientific), and 10% fetal bovine serum (Hyclone). Depending on the experiment, cells were plated with a final concentration of 2 *μ*g/mL polyethylenimine on either 8-well glass slides (CELLTREAT, MidSci), 24-well, 6-well, and 10-cm TPP cell culture plates (MidSci) at densities of 35,000 cells/well, 200,000 cells/well, 600,000 cells/well, and 4.5 million cells, respectively.

As previously outlined, HEK293 cells were transfected using the Promega FuGENE HD protocol (Fugent LLC) 24 hours after plating.^25^ Cells were transfected with empty vector (EV), His- or Flag-tagged versions of wild-type (WT) OATP1B1, variants of OATP1B1, WT OATP1B3, or variants of OATP1B3. Uptake of model substrates was the same for the untagged and the tagged versions of OATP1B1 and OATP1B3 (Supplemental Fig. 1). For 8-well glass slides, one well was transfected per condition with 100 ng of DNA. Three wells were transfected per condition on 24-well plates with 500 ng of DNA. Six-well plates were transfected with 3 *μ*g of DNA, and only one well was used per condition. For 10-cm plates, a total of 15 *μ*g of DNA was used for transfection. All coexpression experiments were transfected with the corresponding plasmid in a 1:1 ratio.

### 2.2 Transporter uptake assays and kinetics

For the treatment with 2-bromopalmitate (2BP; Sigma-Aldrich), 2BP was dissolved in 0.5% methanol. Transporter-mediated uptake was measured under initial linear rate conditions for 20 seconds for 0.1 *μ*M [^3^H]-estradiol-17*β*-glucuronide and 0.1 *μ*M [^3^H]-estrone-3-sulfate, and for 60 seconds for 0.1 *μ*M [^3^H]-rosuvastatin and ^3^[H]-cholecystokinin-8 in transfected HEK293 cells using a previously established and optimized procedure.^25^, ^26^, ^27^ Radiolabeled [^3^H]-estradiol-17*β*-glucuronide (31.2 Ci/mmol), [^3^H]-estrone-3-sulfate (48.9 Ci/mmol), and ^3^[H]-cholecystokinin-8 (113.4 Ci/mmol) were purchased from Perkin Elmer. Estradiol-17*β*-glucuronide sodium salt (E1127) and Estrone 3-sulfate sodium salt (E0251) were purchased from Sigma-Aldrich. Rosuvastatin (12029) was obtained from Cayman Chemicals. Cholecystokinin octapeptide (4033010) was purchased from Bachem.

In summary, cells were washed thrice with 1 mL of warm HEK293 uptake buffer (142 mM NaCl, 5 mM KCl, 1 mM KH_2_PO_4_, 1.2 mM MgSO_4_, 1.5 mM CaCl_2_, 5 mM glucose, 12.5 mM HEPES). Cells were then incubated for 20 or 60 seconds with 200 *μ*L of uptake solution containing the corresponding radioactive solution at 37 °C. After the allotted time, the uptake solution was removed, and the cells were washed 3 times with 1 mL of cold HEK293 uptake buffer. Cells were then lysed using 300 *μ*L 1% TX-100 in PBS and resuspended. Two hundred microliter of the resuspended cell lysate was mixed with 750 *μ*L of OptiPhase HiSafe 3 scintillation cocktail (Revvity), and radioactivity was determined using a beta counter. Fifty *μ*L of the remaining cell lysate was then used to determine total protein via a bicinchoninic acid (BCA) assay (Thermo Fisher Scientific), and those protein values were then used to normalize uptake. The same uptake function assay protocol steps were used to conduct the kinetics in this study. The only difference is that the initial linear rates were first determined, and then a range of concentrations was assessed at the corresponding time point (20 seconds for OATP1B1).

### 2.3 Acyl-resin–assisted capture

The acyl-resin–assisted capture (acyl-RAC) assays were performed using the CAPTUREome *S*-palmitoylated protein kit following the manufacturer’s protocol (Badrilla). In brief, membrane-enriched fractions from cell lysates of two 10-cm plates transfected with the corresponding plasmids were used as starting samples. To obtain these membrane-enriched fractions, cells on a 10-cm plate were washed twice with 4 mL of cold PBS. One milliliter of ice-cold hypotonic homogenization solution (1 mM NaCl, 5 mM Tris-HCl pH 7.5, and 1x protease inhibitor) was added to the 10-cm plate and used to scrape off the cells. This cell solution was transferred to a glass Teflon homogenizer and homogenized with 25 strokes on ice using a handheld drill. The cell homogenate was then centrifuged at 900*g* for 10 minutes at 4 °C; the supernatant was subsequently collected and centrifuged for 20 minutes at 10,000*g* and 4 °C. The resulting pellet was resuspended in 100 *μ*L of hypotonic homogenization solution, and the final protein concentration was determined using the BCA assay. Equal amounts of protein were used for each condition as the starting sample for the acyl-RAC assay. Samples then underwent all steps outlined in the manufacturer’s protocol (treated with thiol blocking reagent, washed, underwent thioester cleavage, captured on thiol-reactive resin, eluted), and then analyzed on western blots.

For the acyl-RAC assay with human liver, samples were obtained through the Cell Isolation Core in the Department of Pharmacology, Toxicology and Therapeutics at the University of Kansas Medical Center, in accordance with an HSC-approved protocol from donor organs of 2 Caucasian males aged 43 (KHH137) and 61 (KHH144) years. About 50 mg of liver tissue was homogenized in 2 mL of hypotonic homogenization solution using a polytron for 30 seconds. The resulting homogenates were centrifuged for 5 minutes at 600*g*, and the resulting supernatant was centrifuged again for 20 minutes at 10,000*g*. The resulting pellets were resuspended in 100 *μ*L of homogenization buffer and used as outlined above.

### 2.4 Site-directed mutagenesis

As previously outlined, the QuikChange Lightning Site-Directed Mutagenesis Kit (Agilent) was used to carry out the mutagenesis reactions.^27^ A previously cloned His-tagged OATP1B1 in the pcDNA5/FRT vector was used as the mutagenesis template.^28^ Primers were ordered from Thermo Fisher Scientific and were designed to flank the codon of interest (Cys24, Cys101, and Cys691) by approximately 20 nucleotides (Table 1). One Shot TOP10 cells (Thermo Fisher Scientific) were used to transform the mutated complementary DNA. Complementary DNA from randomly selected bacterial colonies was isolated using Qiagen Mini-Prep kits and sequenced (Genewiz). The same protocol was used for OATP1B3. A previously cloned Flag-tagged OATP1B3 in the pcDNA5/FRT vector was used as a template,^2^ and the codons of interest were Cys24 and Cys691. Sequenced constructs were analyzed, and those containing the proper amino acid replacements were then transfected into HEK293 cells for further characterization.

**Table 1:** Site-directed mutagenesis primers

Site of Interest	Forward Primer	Reverse Primer
OATP1B1 Cys24→Ala	5′ - AGAATAAGAAAACAAGATAC GCGAATGGATTGAAGATGTTCTT - 3′	5′ - AAGAACATCTTCAATCCATT CGCGTATCTTGTTTTCTTATTCT - 3′
OATP1B1 Cys24→Ser	5′ - AGAATAAGAAAACAAGATAC AGCAATGGATTGAAGATGTTCTT - 3′	5′ - AAGAACATCTTCAATCCATT GCTGTATCTTGTTTTCTTATTCT - 3′
OATP1B1 Cys101→Ala	5′ - CAAAGTTAATTGGAATCGGT GCGTTCATTATGGGAATTGGAGG - 3′	5′ - CCTCCAATTCCCATAATGAA CGCACCGATTCCAATTAACTTTG - 3′
OATP1B1 Cys691→Ala	5′ - GCAGATAGTGAAACACATGC GCATCATCACCATCACCATTAA - 3′	5′ - TTAATGGTGATGGTGATGAT GCGCATGTGTTTCACTATCTGC - 3′
OATP1B3 Cys24→ Ala	5′ - AGAAAAAGAAAACAAGACGC GCCAATGGATTCAAGATGTTCTT - 3′	5′ - AAGAACATCTTGAATCCATT GGCGCGTCTTGTTTTCTTTTTCT - 3′
OATP1B3 Cys24→ Ser	5′ - AGAAAAAGAAAACAAGACGC TCCAATGGATTCAAGATGTTCTT - 3′	5′ - AAGAACATCTTGAATCCATT GGAGCGTCTTGTTTTCTTTTTCT - 3′
OATP1B3 Cys691→Ala	5′ - GCTGGAACAGATAGTAAAAC AGCGAATTTGGACATGCAAGAC - 3′	5′ - GTCTTGCATGTCCAAATTCG CTGTTTTACTATCTGTTCCAGC - 3′

### 2.5 Surface biotinylation assay

HEK293 cells were plated on 6-well plates and transfected using the protocols outlined above. After 48 hours, surface expression was determined using a previously optimized procedure.^27^ First, cells rested on ice for 15 minutes. Each well was then washed twice with 4 mL of ice-cold PBS and incubated while rocking for 1 hour at 4 °C with 2 mL of 1 mg/mL EZ-Link NHS-SS-Biotin in PBS (Thermo Fisher Scientific). After washing cells twice with 4 mL of PBS, they were incubated for 20 minutes with 4 mL of 100 mM glycine in PBS while rocking at 4 °C. Cells were washed again with 4 mL of PBS and then lysed with 1 mL of lysis buffer (150 mM NaCl, 1 mM EDTA, 0.1% SDS, 1% Tx-100, and 10 mM Tris-HCl pH 7.5) for 10 minutes. Lysates were centrifuged for 2 minutes at 10,000*g*, and 150 *μ*L were saved for total protein analysis. The supernatant was incubated at room temperature using end-over-end rotation for 1 hour with 150 *μ*L of Pierce Streptavidin Magnetic beads (Thermo Fisher Scientific) prewashed twice with 500 *μ*L of lysis buffer. Resin beads were then washed 6 times for 5 minutes using end-over-end rotation with 500 *μ*L of lysis buffer. If samples were analyzed via mass spectrometry, the assay was completed with 3 washes of 500 *μ*L of PBS, and the beads were stored at –80 °C until analysis was conducted. For samples analyzed using western blot, the captured surface proteins were then eluted from the beads at 70 °C for 10 minutes with 1x SDS sample buffer containing 5% *β*-mercaptoethanol and 1x of cOmplete Protease Inhibitor (Roche Diagnostics), and the eluates were separated on a 4%–20% gel, transferred to PVDF membranes, and processed as described below. Previous experiments demonstrated that glyceraldehyde-3-phosphate dehydrogenase was not detectable after elution in the surface biotinylated fraction.

### 2.6 Western blotting

Protein samples were heated to 50 °C for 10 minutes before separation on polyacrylamide gels. Then, 4%–20% gradient gels (10-well, 50 *μ*L or 12-well, 20 *μ*L) were purchased from Bio-Rad Laboratories or MP Biomedicals, and 7.5% gels were purchased from Bio-Rad Laboratories or made in-house. After separation, the Mini Trans-Blot system from Bio-Rad was used to transfer proteins to PVDF membranes. Blots were then blocked in 5% milk in Tris-buffered saline/Tween 20 (TBST) (Tris-buffered saline [TBS] and 0.1% Tween-20) for 1 hour at room temperature. Blots were probed with the corresponding primary antibodies in 5% milk in TBST overnight at 4 °C, then washed 5 times for 2 minutes with TBST before room temperature incubation with corresponding horseradish peroxidase–conjugated secondary antibodies in 2.5% milk in TBST for 1 hour. The antibodies used were the mouse Tetra His antibody (34670, 1:2000) from Qiagen. Rabbit anti-His antibody (ab213204, 1:1000) and alpha 1 Sodium Potassium ATPase antibody (ab7671, 1:2000) were purchased from Abcam. Rabbit anti-OATP1B1 antibody (K23, 1:1000) and anti-OATP1B3 (K28, 1:1000) were a generous gift from Dr Bruno Stieger (University Hospital Zurich, Switzerland). Monoclonal anti-Flag M2 mouse antibody (F1804, 1:1000) was purchased from Sigma-Aldrich. Horseradish peroxidase–conjugated goat anti-mouse or rabbit secondary antibodies (31430 or 31460; both 1:10,000) were purchased from Thermo Fisher Scientific. After the antibody incubations, the blots were washed 3 times for 5 minutes each with TBST, followed by a final wash with TBS. Subsequently, the SuperSignal West Pico (34580) or Femto (34095) chemiluminescent substrate was added for 5 minutes. Blots were imaged using the LI-COR Odyssey Fc (LI-COR) and quantified using the Image Studio Lite Quantification Software (version 5.2.5).

### 2.7 Purification of Flag-tagged proteins and proteomic analysis

First, the membrane-enriched fraction of Flag-tagged OATP1B1 was isolated from 10-cm plates of transiently transfected HEK293 cells using the protocol previously outlined in this article. Two hundred microgram of membrane-enriched Flag-tagged OATP1B1 was then incubated at 4 °C using end-over-end rotation for 30 minutes with 1200 *μ*L of solubilization buffer (500 mM NaCl, 50 mM sodium monobasic phosphate pH 8.0, 1x EDTA-free protease inhibitor cocktail, and 1% dodecyl *β*-D-maltoside detergent [DDM]). The solubilized sample was centrifuged at 4 °C at 20,000*g* for 30 minutes. The supernatant was retained and added to 50 *μ*L of anti-Flag M2 Magnetic Beads, which were purchased from Sigma-Aldrich, and prewashed twice with the solubilization buffer. This mixture was incubated at 4 °C using end-over-end rotation overnight. The following day, the magnetic beads were washed twice with 500 *μ*L of wash buffer (500 mM NaCl, 50 mM sodium monobasic phosphate pH 8.0, 1x EDTA-free protease inhibitor cocktail, and 0.1% DDM). The protein was eluted from the beads with 200 *μ*L of elution buffer (1 M glycine, 0.1% DDM, pH 2.8) and then neutralized with 30 *μ*L of neutralization buffer (1 M Tris, pH 8.5). Eluted protein was then transported to the Kansas City Children’s Mercy Hospital, Division of Clinical Pharmacology, Toxicology and Therapeutics Innovation, where the proteomic analysis was performed.

### 2.8 Mass spectrometry

For palmitoylation analysis, purified Flag-tagged OATP1B1 was dialyzed against 50 mM TEAB 0.1% DDM. The sample was reduced with tris(2-carboxyethyl)phosphine, alkylated with iodoacetamide, and digested with trypsin. Peptide analysis was performed on an Exploris 480 Mass Spectrometer (Thermo Fisher Scientific), configured with an EASY-Spray source and coupled to an EASY-nLC 1200 (Thermo Fisher Scientific). The peptides were separated on a 15 cm (2 *μ*m particle size) EASY-Spray column (Thermo Scientific). Mobile phase A was 0.1% formic acid/water, and mobile phase B was 0.1% formic acid/80% acetonitrile/20% water. The gradient increased from 5% B to 65% B at a flow rate of 300 nL/min over 2 hours. The mass spectrometer parameters were as follows: spray voltage 1900 V and ion transfer temperature 280 °C. For data-dependent acquisition runs, the parameters were as follows: precursor resolution 120,000, scan range 400 to 1200 m/z, RF lens 50%, profile mode, normalized AGC target 300%, isolation window 1.4 m/z, HCD collision energy 30%, and MS2 resolution 15,000. The data were searched using the Uniprot canonical human FASTA and Proteome Discoverer 2.4 with palmitoylation (C,K) and carbamidomethylation (C) as variable modifications.

For quantitative analysis of the surface proteome, the washed streptavidin beads were diluted with 20 mM TEAB, followed by reduction with dithiothreitol and alkylation with iodoacetamide. The enriched proteins were on-bead digested with trypsin overnight at 37 °C in 1 M urea in 20 mM TEAB and 1 mM calcium chloride. After digestion, the supernatant and a bead wash (30% acetonitrile) were transferred to a fresh microcentrifuge tube. A nitrogen blower was used to evaporate the acetonitrile, and the sample was passed through a molecular weight cutoff filter.

For the quantitative analysis of the surface proteome, a parallel reaction monitoring (PRM) method was used. A matrix-matched calibration curve was generated as described^29^^,^^30^ by diluting WT transporter-expressing cell lysate in chicken serum at the following percentages: 100%, 30%, 10%, 3%, 1%, and 0.3%. The calibration curve was analyzed in quadruplicate for surface expression studies. The data were acquired on the Exploris 480 as described above, with a few changes. The liquid chromatography gradient increased from 5% B to 40% B over 20 minutes, followed by a 2-minute gradient to 100% B, and hold at 100% B for 8 minutes. For PRM runs, the Exploris parameters were as follows: resolution 60,000, scan range 400–1200 m/z, RF lens 50%, profile mode, isolation window 2 m/z, HCD collision energy 30%, MS2 resolution 15,000. A DDA raw file of the top end of the standard curve was processed using Proteome Discoverer and used to create a spectral library in Skyline 23.1.0.455. PRM data for the samples and calibration curve were extracted using Skyline with the corresponding library files. The peptides scheduled for PRM are summarized in Table 2. Normalization on a per-sample basis was achieved using the summed area of the transitions for cadherin-2 (CADH2), integrin *β*-1 (ITB1), and sodium/potassium transporting ATPase subunit *α* (AT1A1).

**Table 2:** Proteins and peptides for quantitative surface proteome analysis

Protein	Peptide	m/z	z	Analyte (A) or Normalization (N)
sp|Q9NPD5|SO1B3_HUMAN	ALAMGFQSMVIR	662.35	2	A
sp|Q9NPD5|SO1B3_HUMAN	IYNSVFFGR	551.79	2	A
sp|Q9NPD5|SO1B3_HUMAN	VMDEANLEFLNNGEHFVPSAGTDSK	907.75	3	A
sp|P05023|AT1A1_HUMAN	AVFQANQENLPILK	792.94	2	N
sp|P05023|AT1A1_HUMAN	AVAGDASESALLK	616.33	2	N
sp|P05023|AT1A1_HUMAN	IVEIPFNSTNK	631.34	2	N
sp|P05023|AT1A1_HUMAN	NPNTSEPQHLLVMK	536.61	3	N
sp|P19022|CADH2_HUMAN	IDPVNGQITTIAVLDR	862.98	2	N
sp|P19022|CADH2_HUMAN	LNGDFAQLNLK	616.84	2	N
sp|P19022|CADH2_HUMAN	FLEAGIYEVPIIITDSGNPPK	1137.11	2	N
sp|P19022|CADH2_HUMAN	SAAPHPGDIGDFINEGLK	613.31	3	N
sp|P05556|ITB1_HUMAN	LKPEDITQIQPQQLVLR	673.73	3	N
sp|P05556|ITB1_HUMAN	LSENNIQTIFAVTEEFQPVYK	824.09	3	N
sp|P05556|ITB1_HUMAN	LSEGVTISYK	548.80	2	N
sp|Q9Y6L6|SO1B1_HUMAN	NVTGFFQSFK	587.80	2	A
sp|Q9Y6L6|SO1B1_HUMAN	SVAGLTMTYDGNNPVTSHR	673.99	3	A
sp|Q9Y6L6|SO1B1_HUMAN	VYLGLSSMLR	569.82	2	A

AT1A1, ATPase subunit *α*; CADH2, cadherin-2; ITB1, integrin *β*-1.

### 2.9 Proximity ligation assay

Interactions between OATP1B1 and OATP1B3 were detected using the Naveni TriFlex Cell kit, purchased from Navinci, according to the manufacturer’s protocol. In summary, cells plated on 8-well glass slides were fixed for 10 minutes with ice-cold methanol, washed twice with PBS, and then permeabilized with 0.3% TritonX-100 in PBS for 10 minutes. After blocking with the kit-provided blocking buffer (components and concentrations are proprietary), cells were incubated overnight at 4 °C with the corresponding primary antibody. His-tagged proteins were detected using rabbit anti-His antibody (ab213204, 1:1000), while Flag-tagged proteins were detected with monoclonal anti-Flag M2 mouse antibody (F1804, 1:1000). All subsequent steps were followed per the manufacturer’s protocol. Slides were mounted using the Vectashield antifade mounting medium with DAPI (H-1200) (VectorLabs). Slides were imaged on a Nikon CSU-W1 spinning-disk confocal microscope with SoRa. Quantifications were conducted in Fiji (Version 2.14.0/1.54f) as follows.^31^ Three pictures were used per condition per experiment, and the experiments were repeated at least 3 times. The area of each yellow punctum was quantified and normalized to the total protein signal and number of cells in the image using Fiji as follows. The area of the signal (either green, red, or yellow) was divided by the number of cells (DAPI) to obtain the individual ratios. The number obtained from the EV + EV control pictures was used as background and subtracted from the numbers calculated for the OATP pictures. The final expression levels were calculated by dividing the interaction signal (yellow ratio) by the total protein signal (green ratio plus red ratio) and finally converted to a percentage of control for easier visualization. The percentage of control was then calculated from 3 independent experiments to compare signals with the coexpression of WT OATP1B1 + OATP1B3. In separate experiments, the expression of different proteins under different conditions was verified using immunofluorescence with the individual antibodies.

### 2.10 Statistical analysis

One-way ANOVA was used to analyze data for significant differences between groups, followed by “Dunnett’s multiple comparisons tests” in GraphPad Prism 10 (GraphPad Software Inc). Significance was determined by a *P* value less than .05.

## 3 Results

### 3.1 Effect of 2BP on OATP1B1 uptake function and surface expression

**Fig. 1:**
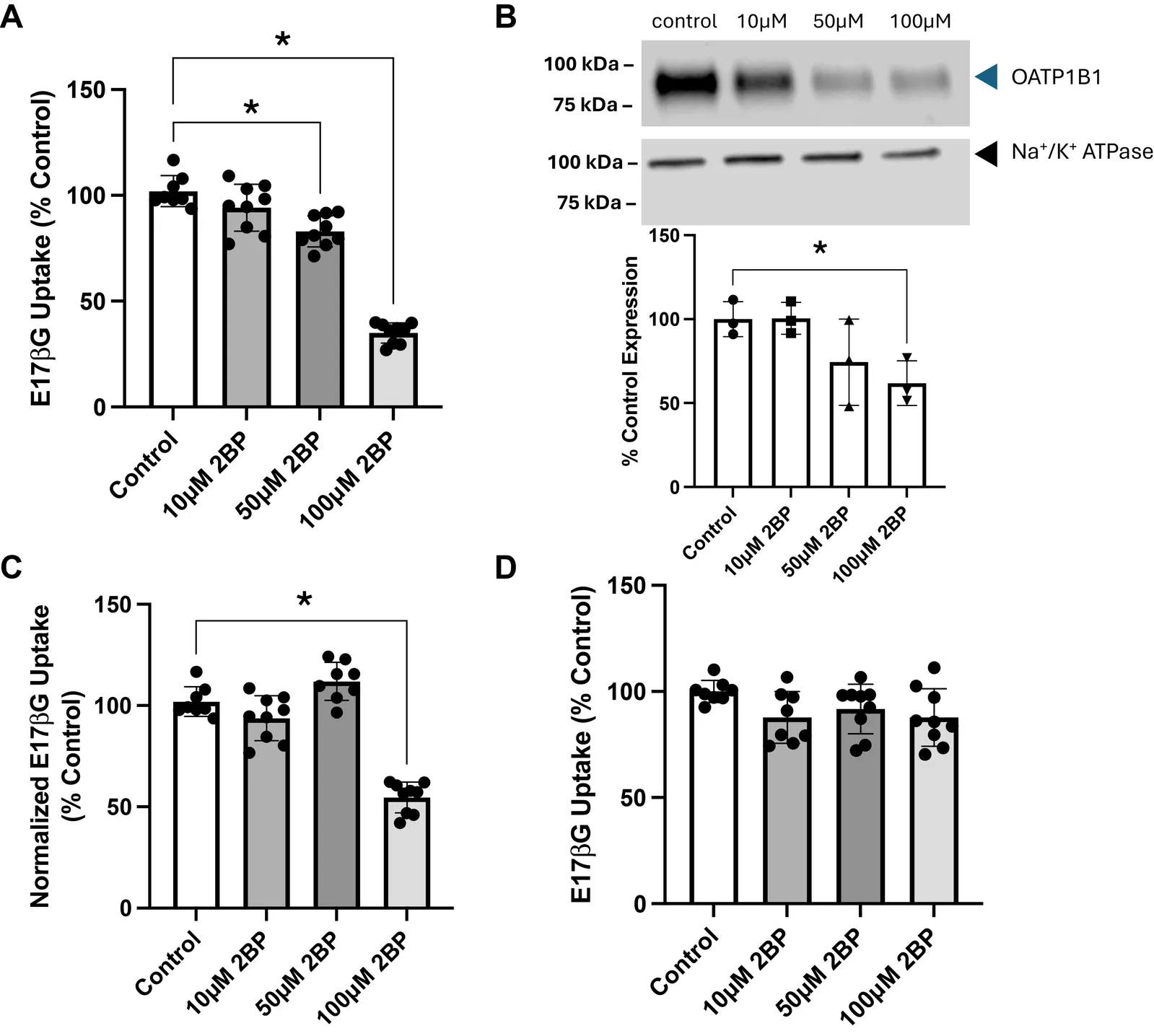
Effect of 2BP on OATP1B1 uptake function and surface expression. (A) Uptake of 0.1 *μ*M [^3^H]-estradiol-17*β*-glucuronide (E17*β*G; 0.3 *μ*Ci/mL of radioactivity) in OATP1B1 transiently transfected HEK293 cells was measured 48 hours after transfection for 20 seconds (initial linear rate conditions) at 37 °C after 18-hour treatment with increasing concentrations of 2BP (dissolved in methanol). The control was treated with methanol at a final concentration of 0.5% to match the methanol concentration of the 2BP-treated cells. Net uptake was calculated by subtracting the uptake of EV-transfected cells from OATP1B1-transfected cells. (B) A representative western blot of surface-expressed OATP1B1 after 18-hour treatment with increasing concentrations of 2BP is shown. His-tagged OATP1B1 was detected with the Tetra His antibody; quantification of expression relative to the control and the loading control (Na^+^/K^+^ ATPase, detected with an antibody against the *α* 1 subunit) is shown below the blot (mean ± SD; *n* = 3). (C) Uptake results normalized for surface expression. (D) Uptake of 0.1 *μ*M [^3^H]-E17*β*G (0.3 *μ*Ci/mL of radioactivity) in HEK293 cells transiently transfected with OATP1B1 was measured for 20 seconds in the absence and presence of increasing 2BP concentrations. All uptake results are shown as a percentage of the control and are reported as the mean ± SD of 3 independently conducted experiments. Statistical significance was analyzed using one-way ANOVA in GraphPad Prism 10; asterisks indicate *P* < .05.

At the beginning of this study, we used 2BP, a nonselective inhibitor of *S*-palmitoylation that is widely used in the literature, as a starting point to investigate *S*-palmitoylation in OATP1B1. We observed a dose-dependent decrease in the uptake function of OATP1B1-mediated uptake after 18-hour treatment with increasing concentrations (10 *μ*M, 50 *μ*M, and 100 *μ*M) of 2BP (Fig. 1A). We then measured the plasma membrane expression of OATP1B1 after the same 18-hour treatment with 2BP to determine if the decrease in function could be attributed to a reduction in surface expression. At 100 *μ*M 2BP, plasma membrane expression of OATP1B1 decreased by about 40% (Fig. 1B). The quantified surface expression was then used to normalize the uptake function and demonstrated that there was still a decrease in the uptake function of OATP1B1 treated with 100 *μ*M 2BP, which is not a direct result of surface expression changes (Fig. 1C). Acute treatment of OATP1B1 with increasing 2BP concentrations did not impact its uptake function, suggesting that 2BP is not a direct inhibitor of OATP1B1 (Fig. 1D). Overall, these results suggest that OATP1B1 may be *S*-palmitoylated; however, due to the nonselective nature of this inhibitor, a more direct method was needed to corroborate this.

### 3.2 OATP1B1 contains S-palmitoylated residues

**Fig. 2:**
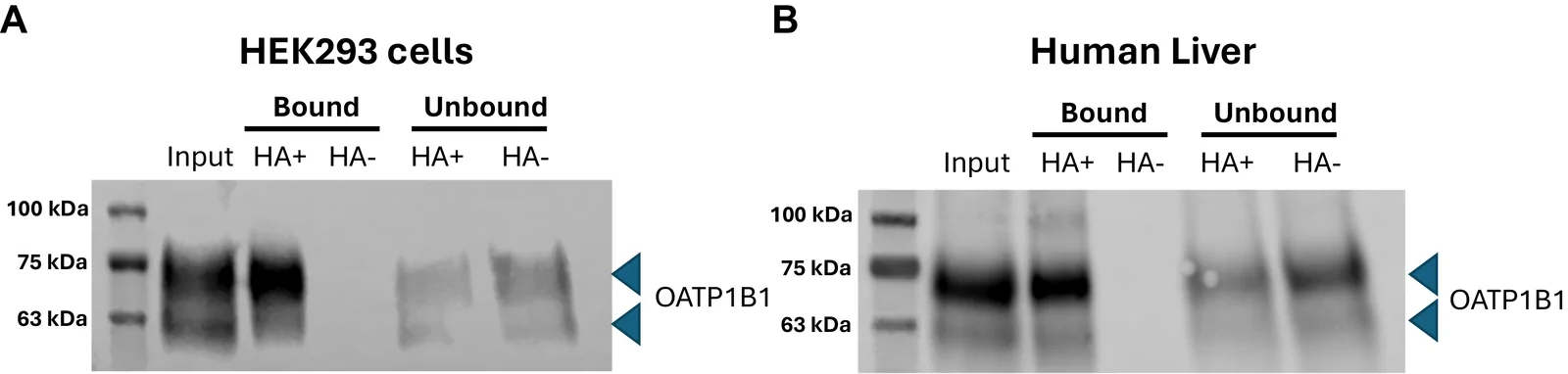
Acyl-resin–assisted capture (RAC) assay of OATP1B1. (A) Representative western blot of the acyl-RAC assay conducted with HEK293 cells transiently transfected with His-tagged OATP1B1 and probed with the Tetra His antibody. (B) Western blot of acyl-RAC analysis of a human liver sample (KHH137). Native OATP1B1 was detected with the K23 antibody. The acyl-RAC assay (Supplemental Fig. 2) was performed with a membrane-enriched fraction of HEK293 cells expressing His-tagged OATP1B1 or with a membrane-enriched fraction of a human liver sample (see Materials and Methods). After treating the samples with *N*-ethylmaleimide to block all free SH groups, the sample was split into 2 fractions. One fraction was treated with hydroxylamine (HA+) to cleave the palmitoyl-thioester bond generating a free sulfhydryl, which was then captured with a thiopropyl sepharose resin. The other fraction was treated with the “Acyl Preservation Reagent” (CAPTUREome kit) (HA-), which does not cleave the palmitoyl-thioester bond and thus does not bind to the resin. After centrifugation at 16,000*g*, the supernatant was kept as “Unbound” samples, and the resin was washed 5 times with binding buffer. After the last wash, captured proteins (“Bound” samples) were eluted with 2x Laemmli sample buffer, heated to 60 °C for 10 minutes, separated on a 4% to 20 % gel, and OATP1B1 was detected after transfer to PVDF membranes as described in Materials and Methods.

To directly verify that OATP1B1 is *S*-palmitoylated, we performed the acyl-RAC assay on HEK293 cells transiently transfected with OATP1B1 using the Badrilla CAPTUREome *S*-Palmitoylated Protein Kit. First, free thiol groups were blocked. Secondly, thioester linkages were cleaved with hydroxylamine (HA), which allowed the liberated thiol groups to be captured on a thiol-reactive resin. Proteins were then eluted and ultimately analyzed by western blotting (Supplemental Fig. 2). This assay shows that treatment with HA (HA+) results in OATP1B1 binding to the resin, directly demonstrating that *S*-acylation residues are present in this hepatic transporter (Fig. 2A). Similar results were observed when this assay was conducted on human liver samples (Fig. 2B), demonstrating that *S*-palmitoylated residues were present in OATP1B1.

### 3.3 Identifying palmitoylation residues in OATP1B1

**Fig. 3:**
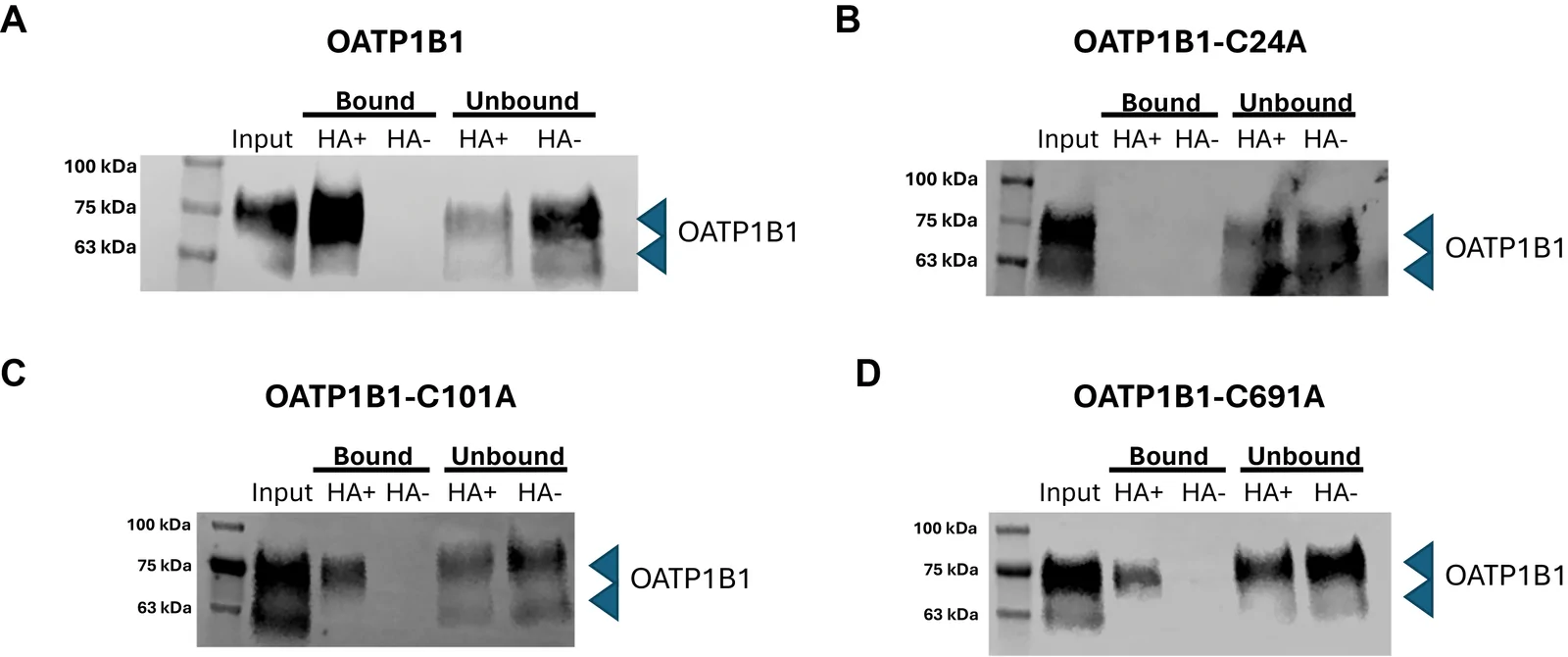
Acyl-RAC assay of OATP1B1 and 3 mutants. Potential *S*-palmitoylation residues (cysteines) of OATP1B1 were mutated to alanine residues. Wild-type OATP1B1 and the resulting mutants were transiently expressed in HEK293 cells and analyzed using the Badrilla CAPTUREome *S*-Palmitoylated Protein Kit. Representative western blots of this assay show that wild-type OATP1B1 (A), OATP1B1-C101A (C), and OATP1B1-C691A (D) were similarly *S*-acylated, whereas OATP1B1-C24A was not *S*-acylated (B). HA-, samples were treated with Tris; HA+, samples were treated with hydroxylamine. For experimental details, see legend to Fig. 2 and Supplemental Fig. 2.

GPS-Palm, an *S*-palmitoylation site prediction software, was used to identify potential palmitoylation sites in OATP1B1.^32^ Unfortunately, there are no consensus sequences known, so far, to accurately predict palmitoylation sites. Among the predicted palmitoylation sites, Cys24, Cys101, and Cys691 were the most likely to be modified because they are either in the cytoplasmic N or C termini (Cys24 and Cys691) or in the cytoplasmic half of transmembrane domain 3. All other predicted *S*-palmitoylation sites were in extracellular loops and, thus, not further considered. These sites were mutated to alanine residues using site-directed mutagenesis and analyzed through acyl-RAC assays (Fig. 3). This assay identified that OATP1B1’s Cys24 site was *S*-palmitoylated, as only when Cys24 was mutated to Ala24 did OATP1B1 lose its *S*-palmitoylation (Fig. 3B).

### 3.4 Characterizing uptake function and surface expression of nonpalmitoylated OATP1B1

**Fig. 4:**
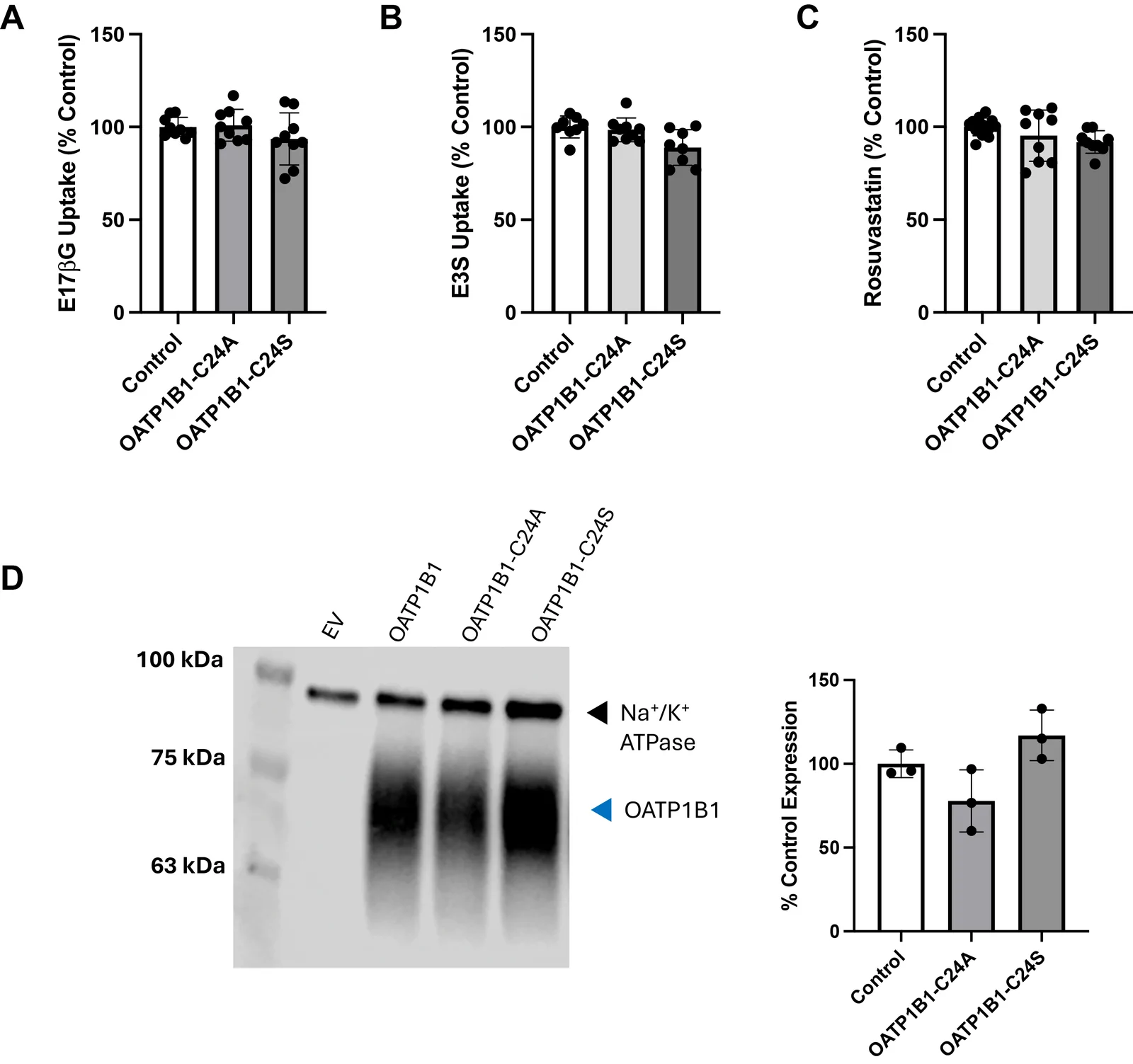
Uptake and surface expression of OATP1B1 Cys24 mutants. Alanine or serine mutants of cysteine at position 24 (C24A or C24S) were transiently expressed in HEK293 cells, and 48 hours later, substrate uptake was measured. (A) Twenty-second uptake of 0.1 *μ*M [^3^H]-estradiol-17*β*-glucuronide (E17*β*G; 0.3 *μ*Ci/mL of radioactivity), (B) 20-second uptake of 0.1 *μ*M [^3^H]-estrone-3-sulfate (E3S; 0.3 *μ*Ci/mL of radioactivity), and (C) 1-minute uptake of 0.1 *μ*M [^3^H]-rosuvastatin (0.3 *μ*Ci/mL of radioactivity) was measured at 37 °C. Net uptake was calculated by subtracting the uptake of EV-transfected cells from that of mutant-transfected cells. (D) Representative western blot of surface-expressed wild-type and mutant His-tagged OATP1B1, along with the loading control (Na^+^/K^+^ ATPase, detected with an antibody against the *α* 1 subunit) (left) and quantification of expression relative to the control (right). All uptake results are shown as a percentage of the control, and the means ± SD of 3 independent experiments were quantified.

To test the consequences of mutating the cysteine at position 24 to an alanine, we measured uptake of estradiol-17*β*-glucuronide, estrone-3-sulfate, and rosuvastatin (Fig. 4, A–C). We did not observe any change in function for C24A when compared to OATP1B1. To rule out that this was only true for the alanine substitution, we also replaced the cysteine with a serine and tested it in parallel. Again, similar uptake results were observed compared to WT OATP1B1 (Fig. 4, A–C). The surface expression of these mutants was also investigated using surface biotinylation; no significant changes were observed when compared to WT OATP1B1 (Fig. 4D).

To further characterize the potential effects of palmitoylation on transport, the kinetics of OATP1B1-mediated uptake of estradiol-17*β*-glucuronide and estrone-3-sulfate by the Cys24 mutants were determined. Table 3 summarizes the respective *K*_m_, *V*_max_, and *V*_max_/*K*_m_ values for WT OATP1B1, OATP1B1-C24A, and OATP1B1-C24S. For estradiol-17*β*-glucuronide uptake, there was no significant change in any kinetic parameters between WT OATP1B1 and the Cys24 mutants. Kinetics for estrone-3-sulfate uptake showed a slight decrease in the *V*_max_ for the Cys24 mutants compared to WT, along with a slight increase in capacity (*V*_max_/*K*_m_), but overall, there was no significant change.

**Table 3:** Kinetic parameters of OATP1B1 and Cys24 mutants Kinetics of estradiol-17*β*-glucuronide and estrone-3-sulfate mediated uptake by OATP1B1 wild-type and mutants were measured under initial linear rates at 20 seconds. After subtracting uptake into empty vector–transfected cells, kinetic parameters (*V*_max_ and *K*_m_) were calculated using the Michaelis-Menten equation in GraphPad Prism 10. The results are reported as mean ± SEM of at least 3 independent experiments performed in triplicate.

Substrate	Wild-type	OATP1B1-C24A	OATP1B1-C24S
Estradiol-17*β*-glucuronide			
*K*_m_ (*μ*M)	8.6 ± 1.3	6.6 ± 0.7	9.2 ± 1.1
*V*_max_ (pmol/mg/min)	377.7 ± 19.5	354.6 ± 10.7	391.9 ± 15.8
*V*_max_/*K*_m_ (*μ*L/mg/min)	45.6 ± 5.8	54.0 ± 4.6	45.4 ± 7.6
Estrone-3-sulfate			
*K*_m_ (*μ*M)	1.1 ± 0.2	0.7 ± 0.1	0.6 ± 0.1
*V*_max_ (pmol/mg/min)	183.2 ± 17.4	133.0 ± 9.6	132.3 ± 7.4
*V*_max_/*K*_m_ (*μ*L/mg/min)	168.2 ± 25.8	222.4 ± 2.2	223.1 ± 25.8

### 3.5 Identifying and characterizing the OATP1B3 S-palmitoylation site

**Fig. 5:**
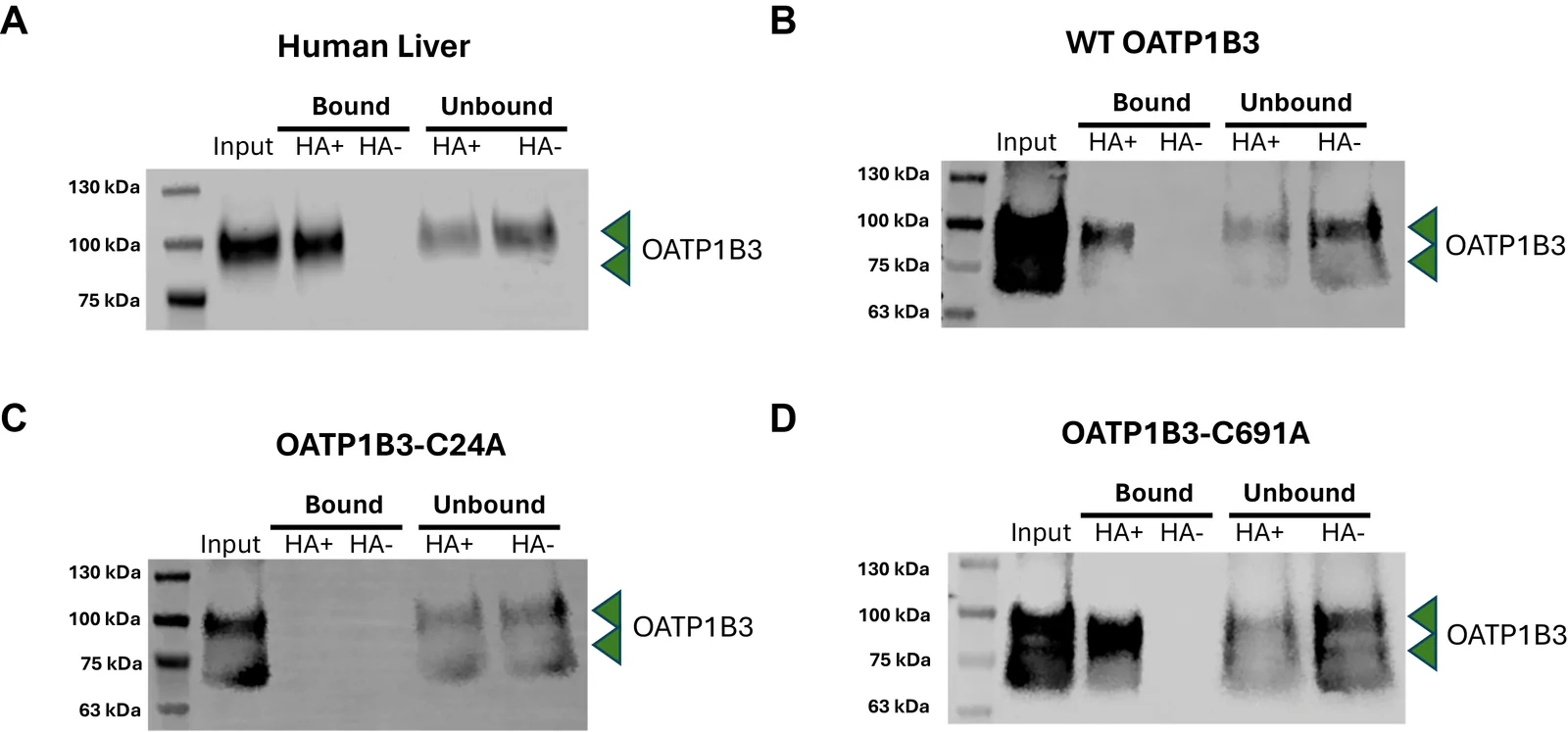
Acyl-RAC assay of OATP1B3 and 2 mutants. The Badrilla CAPTUREome *S*-Palmitoylated Protein Kit confirmed that OATP1B3 is palmitoylated in human liver (KHH144) using the K28 antibody (A) and when transiently expressed in HEK293 cells (B). Potential *S*-palmitoylation residues of OATP1B3 predicted by GPS-Palm were mutated from cysteine to alanine and analyzed using the same kit. OATP1B3-C24A (C) was not palmitoylated, but OATP1B3-C691A (D) was still palmitoylated. Representative western blots are shown. HA-, samples were treated with Tris; HA+, samples were treated with hydroxylamine. For experimental details, see legend to Fig. 2 and Supplemental Fig. 1.

Due to the high amino acid sequence homology between OATP1B1 and OATP1B3, we aimed to determine if OATP1B3 was also *S*-palmitoylated. Acyl-RAC analysis was conducted on human liver samples, and *S*-acylation was observed (Fig. 5A). Residues Cys24 and Cys691 were mutated to Ala using site-directed mutagenesis and underwent acyl-RAC analysis. We observed that, like OATP1B1, OATP1B3 is also palmitoylated at residue Cys24 (Fig. 5, B–D).

**Fig. 6:**
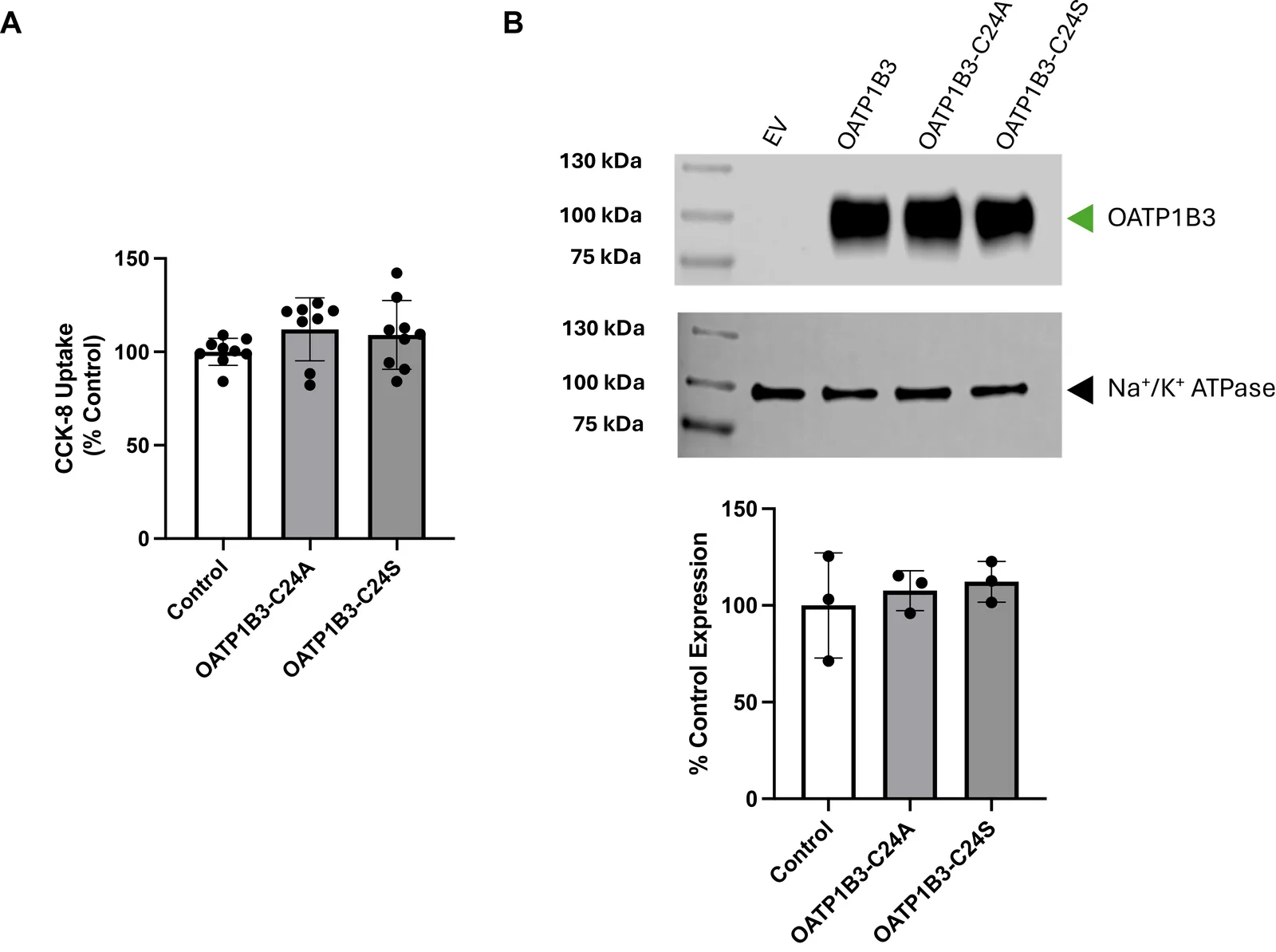
Uptake and surface expression of nonpalmitoylated OATP1B3. OATP1B3-C24A and OATP1B3-C24S, along with OATP1B3 wild type were transiently expressed in HEK293 cells, and 48 hours later, (A) uptake of 0.1 *μ*M [^3^H]-cholecystokinin-8 (CCK-8; 0.3 *μ*Ci/mL of radioactivity) was measured for 1 minute at 37 °C. All uptake results are shown as a percentage of the control and are reported as the mean ± SD of 3 independently conducted experiments. (B) A representative western blot of the surface expressed His-tagged OATP1B3 mutants is shown (top), with quantification of expression relative to control and corrected for expression of the loading control (Na^+^/K^+^ ATPase; detected with an antibody against the *α* 1 subunit) is shown below (mean ± SD; *n* = 3).

The uptake function of OATP1B3 Cys24 mutants (OATP1B3-C24A and OATP1B3-C24S) was assessed by measuring cholecystokinin-8 (CCK-8) uptake. Similar to OATP1B1, no change in uptake function was observed when *S*-palmitoylation was eliminated in OATP1B3 (Fig. 6A). Surface expression of OATP1B3 WT and its palmitoylation mutants showed similar expression levels at the plasma membrane (Fig. 6B).

### 3.6 Characterizing the effect of S-palmitoylation on the function and surface expression of coexpressed OATP1B1 and OATP1B3

Due to the known protein-protein interactions between OATP1B1 and OATP1B3, we aimed to investigate whether *S*-palmitoylation impacts their function and surface expression when coexpressed. In these coexpression experiments, combinations of WT or nonpalmitoylated (C24A version) OATP1B3 and OATP1B1 were assessed and compared to WT function and surface expression levels, which allowed potential palmitoylation-specific changes to be observed. Due to the lack of difference in function between the C24A and C24S versions of OATP1B1 and OATP1B3, only the C24A mutation was used in the following experiments.

**Fig. 7:**
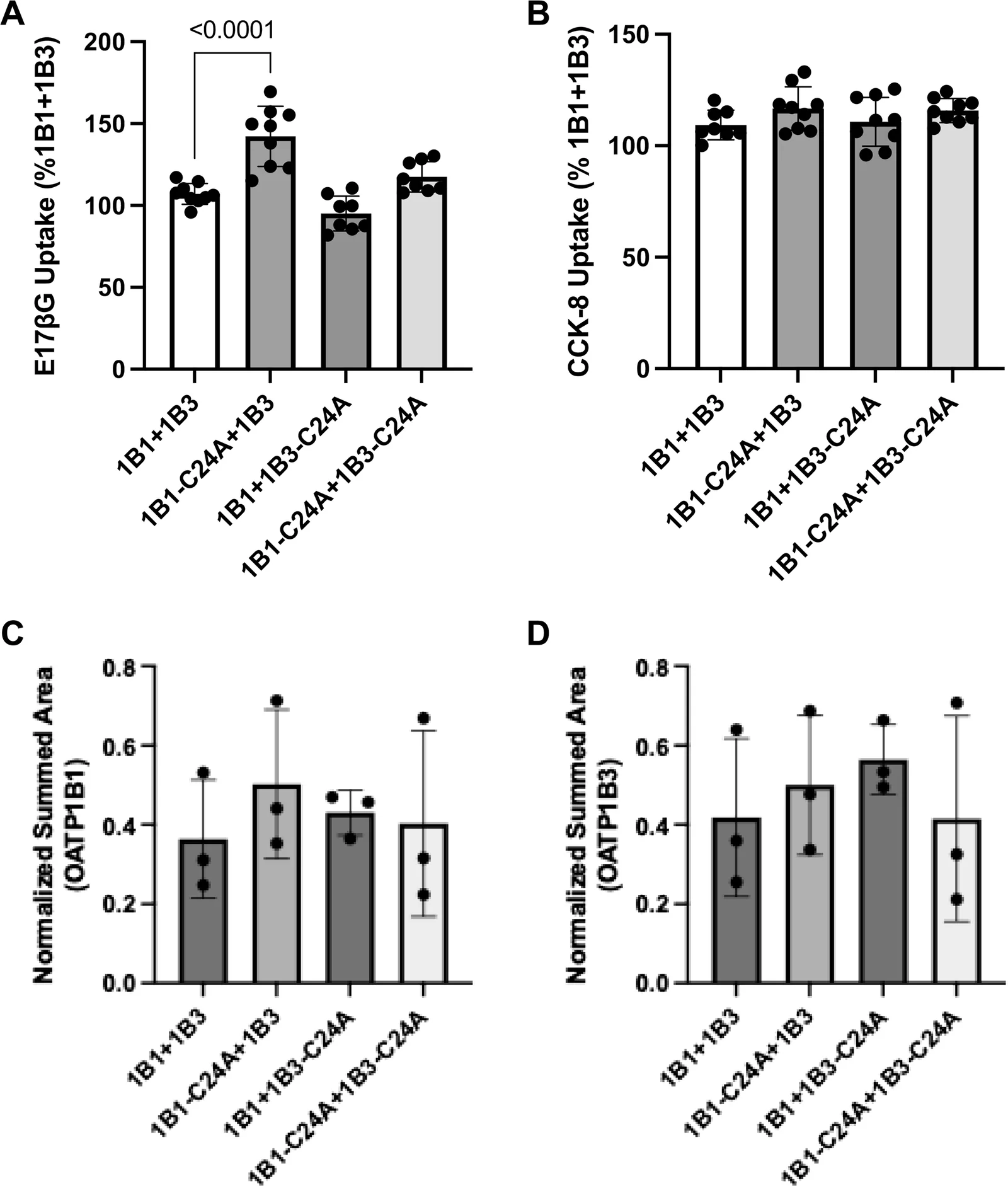
Effect of *S*-palmitoylation when OATP1B1 and OATP1B3 are coexpressed. His-tagged OATP1B1 or His-tagged nonpalmitoylated OATP1B1-C24A were transiently coexpressed with Flag-tagged OATP1B3 or Flag-tagged nonpalmitoylated OATP1B3-C24A. Forty-eight hours after transfection with equivalent (500ng) amounts of each cDNA, (A) 20-second uptake of 0.1*μ*M [^3^H]-estradiol-17*β*-glucuronide (E17*β*G; 0.3 *μ*Ci/mL of radioactivity), or (B) 1-minute uptake of 0.1 *μ*M [^3^H]-cholecystokinin-8 (CCK-8; 0.3 *μ*Ci/mL of radioactivity) was measured at 37 °C. For easier visual representation, “OATP” is omitted from the *x*-axis titles. All uptake results are shown as a percentage of the control and are reported as the mean ± SD of 3 independently conducted experiments. Plasma membrane expression of (C) OATP1B1 and (D) OATP1B3 in each coexpression group was assessed via surface biotinylation and quantified using mass spectrometry. The expression results are the mean ± SD of 3 independent experiments. Statistical significance was analyzed using one-way ANOVA in GraphPad Prism 10.

A slight increase in estradiol-17*β*-glucuronide uptake was observed when OATP1B3 was not palmitoylated (Fig. 7A). No changes in OATP1B3-mediated CCK-8 uptake were observed when OATP1B1’s palmitoylation state was altered (Fig. 7B). Surface biotinylation was conducted on these coexpressed groups, and the results were quantified using mass spectrometry. These results highlight that there is no notable change in surface expression when palmitoylation is altered in any one of the groups (Fig. 7, C and D).

### 3.7 Assessing changes in protein-protein interactions when S-palmitoylation is inhibited in coexpressed OATP1B1 and OATP1B3 via proximity ligation assay

**Fig. 8:**
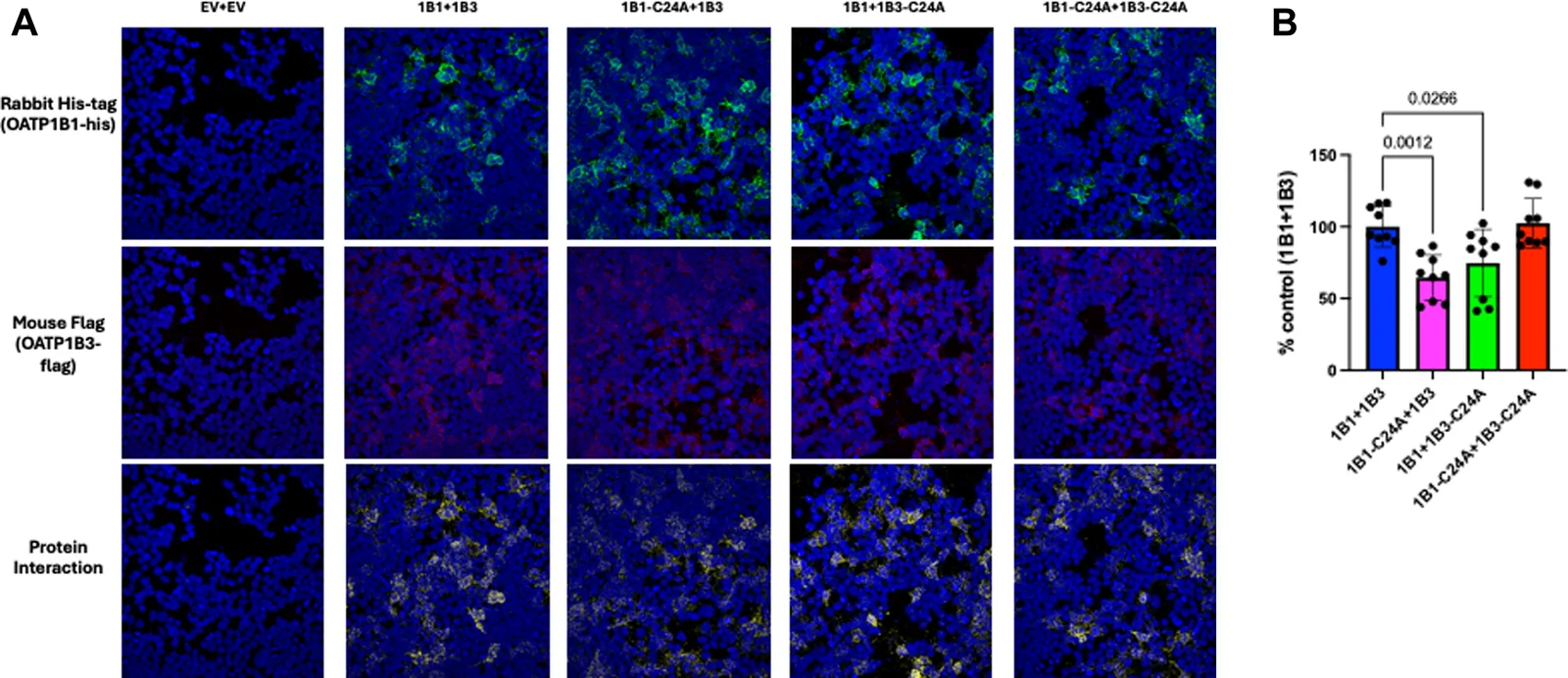
TriFlex protein proximity ligation assay of coexpression of OATP1B1 with OATP1B3. HEK293 cells (35,000 cells/well) were plated on 8-well glass slides and 24 hours later, transfected with the indicated combinations of pcDNA5/FRT EV, His-tagged OATP1B1, His-tagged OATP1B1-C24A, Flag-tagged OATP1B3, and Flag-tagged OATP1B3-C24A (100 ng per plasmid). Forty-eight hours after transfection, cells were fixed, permeabilized with 0.3% TritonX-100 in PBS, incubated with kit-provided blocking buffer, and incubated overnight at 4 °C with the corresponding primary antibodies: His-tagged proteins with rabbit anti-His antibody, Flag-tagged proteins with monoclonal anti-Flag M2 mouse antibody. All subsequent steps were according to the manufacturer’s protocol. Slides were mounted using the Vectashield antifade mounting medium with DAPI and imaged on a Nikon CSU-W1 spinning-disk confocal microscope with SoRa. Signals for individual proteins were detected in this assay; green is the His-tagged protein, and red is the Flag-tagged protein. Protein-protein interactions (proteins are <40 nm apart) are shown as yellow fluorescent puncta. Three pictures were used per condition per experiment, and the experiments were repeated at least 3 times. The area of each yellow punctum was quantified and normalized to the total protein signal and number of cells in the image using Fiji as follows. The area of the signal (either green, red, or yellow) was divided by the number of cells (DAPI) to obtain the individual ratios. The number obtained from the EV + EV control pictures was used as background and subtracted from the numbers calculated for the OATP pictures. The final expression levels were calculated by dividing the interaction signal (yellow ratio) by the total protein signal (green ratio plus red ratio) and finally converted to the percentage of control for easier visualization. The percentage of control was then calculated from 3 independent experiments to compare signals with the coexpression of wild-type OATP1B1 + OATP1B3.

The Naveni TriFlex Cell proximity ligation assay from Navinci can detect individual proteins as well as interactions between 2 proteins through amplified fluorescent signals from oligonucleotide-antibody conjugates. Figure 8 shows a representative result for the detection of His-tagged OATP1B1 (in green), Flag-tagged OATP1B3 (in red), and the interaction between the 2 (in yellow). After quantification of the results, we observed that when either nonpalmitoylated OATP1B1 or nonpalmitoylated OATP1B3 was coexpressed with WT OATP1B3 or WT OATP1B1, there was a significant decrease in the number of yellow signals. This suggests that the protein-protein interaction between OATP1B1 + OATP1B3 is disrupted when the palmitoylation state of one protein is changed. Interestingly, when both nonpalmitoylated proteins are coexpressed, the decrease in signal is restored to control levels (Fig. 8).

## 4 Discussion

*S*-palmitoylation is a reversible PTM that impacts the function and expression of various transporters.^16^^,^^18^^,^^23^^,^^33^^,^^34^ This study aimed to determine if the hepatic drug transporters OATP1B1 and OATP1B3 are palmitoylated, and if this PTM impacts their overall function, surface expression, and protein-protein interaction. We discovered that although both OATP1B1 and OATP1B3 are *S*-palmitoylated, this PTM does not affect their individual function or surface expression. Instead, we observed effects on OATP1B1-OATP1B3 interactions only when the nonpalmitoylation state of one of these proteins was altered.

Using acyl-RAC analysis, we first observed that there are *S*-palmitoylated residues present in OATP1B1 in both transiently transfected HEK293 cells and human liver samples due to the protein signal observed in the HA+ bound lane (Fig. 2). These results also indicated that not all OATP1B1 is palmitoylated at the same time, as demonstrated by the protein signal observed in the unbound HA+ lane; this is not surprising due to the reversible nature of this PTM (Fig. 2). An alternative but less likely explanation given that we used a commercially available and optimized kit, is that the binding to the beads is not 100% efficient or the beads are saturated. Site-directed mutagenesis allowed the identification of Cys24 as the palmitoylation residue in OATP1B1 (Fig. 3). Similar to OATP1B1, we also discovered that OATP1B3 is *S*-palmitoylated at the conserved residue Cys24 (Fig. 5).

Functional and surface expression studies were conducted on OATP1B1 and OATP1B3 site-directed mutants, C24A and C24S versions, which cannot be palmitoylated due to the mutation of the palmitoylation site. Overall, no significant changes in function and surface expression were observed (Figs. 4 and 6). This suggests that *S*-palmitoylation in OATP1B1 and OATP1B3 does not impact the individual uptake function and surface expression of these hepatic transporters.

OATP1B1 and OATP1B3 can form hetero-oligomers that can alter their function and expression.^2^^,^^3^ We wanted to determine if *S*-palmitoylation impacts OATP1B1 and OATP1B3 protein-protein interactions via their coexpression function and surface expression. We observed that when these 2 transporters are coexpressed, there is a slight increase in E17*β*G uptake when nonpalmitoylated OATP1B1 is coexpressed with WT OATP1B3 (Fig. 7A). Interestingly, this increase in function is not observed when nonpalmitoylated OATP1B3 is coexpressed with WT OATP1B1, or when both nonpalmitoylated mutants are coexpressed (OATP1B1-C24A + OATP1B3-C24A) (Fig. 7A). Furthermore, it was not observed when nonpalmitoylated OATP1B1 was expressed alone (Fig. 4A). There are 2 possible explanations: (1) the increased uptake shown in Fig. 7 is due to added uptake by the coexpressed OATP1B3, or (2) the increased OATP1B1-mediated uptake is due to a change in its conformation due to coexpression with OATP1B3 that leads to the increased uptake. Future experiments are needed to clarify which of the 2 explanations is correct. Because no change in OATP1B3 uptake function was observed in any of the coexpression groups (Fig. 7B), it is more likely that the coexpression leads to increased OATP1B1 function, which in turn could result in increased or decreased drug uptake when the 2 proteins are interacting. When investigating coexpression surface membrane localization changes via surface biotinylation, we did not observe a notable difference in surface expression for either OATP1B1 or OATP1B3 (Fig. 7, C and D). These results suggest that palmitoylation of OATP1B1 could play a regulatory role in E17*β*G-mediated uptake when it is coexpressed with OATP1B3.

The proximity ligation assay further explored protein-protein interactions between these 2 transporters when *S*-palmitoylation is disrupted. The fluorescent signal of the interactions was decreased when either nonpalmitoylated OATP1B1 or nonpalmitoylated OATP1B3 was coexpressed with WT OATP1B3 or WT OATP1B1 (Fig. 8). This decrease in interaction is no longer observed when both proteins are nonpalmitoylated (OATP1B1-C24A + OATP1B3-C24A). This suggests that both proteins must reside in the same submembrane domains, either in lipid rafts in their palmitoylated state, or outside of rafts in their nonpalmitoylated state. In either situation, only proteins that are in the same membrane domains would interact with each other. In 2008, the group of Allan Wolkoff determined that rat and mouse OATP1A1 interacted via their C-terminal end, which contains a PDZ recognition sequence with PDZK1, allowing their trafficking to the plasma membrane.^35^ In PDZK1 knockout mice, OATP1A1 accumulated intracellularly, and its expression at the plasma membrane was reduced.^36^ In a recent study, the same group found that OATP1B1 also interacts with PDZK1 and that this interaction is required for plasma membrane expression of OATP1B1.^37^ They even suggested that OATP1B3 would only be trafficked to the plasma membrane when interacting with OATP1B1. Thus, our findings that the palmitoylation status of the 2 proteins can affect their interaction might suggest that these interactions occur well before the proteins are expressed at the plasma membrane, and future experiments in our laboratory are aimed at investigating the trafficking of the 2 proteins, WT and palmitoylation mutants, from the endoplasmic reticulum to the plasma membrane. We hypothesize that the palmitoylation status not only affects the distribution of these 2 OATPs to lipid rafts but also their trafficking to the plasma membrane.

Another interesting component of this study was unearthed early in our work with 2BP, a widely used *S*-palmitoylation inhibitor (Fig. 1). It is important to note that although 2BP is commonly used as an inhibitor of *S*-palmitoylation, studies have shown that it has off-target effects.^38^^,^^39^ Therefore, we wanted to determine if the dose-dependent decrease in uptake function we observed after treatment of WT OATP1B1 with 2BP treatment was directly due to *S*-palmitoylation changes. We treated OATP1B1 nonpalmitoylated mutants with 2BP for 18 hours and observed that both OATP1B1-C24A and OATP1B1-C24S exhibit similar dose-dependent decrease in uptake function as WT OATP1B1 (Supplemental Fig. 3). These results demonstrate that 2BP alters the function of OATP1B1 in an off-target mechanism that does not directly correspond to its palmitoylation state and could mean that regulators of the 2 proteins are palmitoylated and will affect the expression and function of OATP1B1 and OATP1B3 in a palmitoylation-dependent way.

Because OATP1B1 and OATP1B3 are important hepatic drug transporters with a broad substrate affinity for various therapeutic agents and play an essential role in drug disposition, a better understanding of how different modifications impact their function and expression can be crucial in successful patient care. Recent reports demonstrate that obesity can influence palmitoylation and, in turn, affect lipid metabolism and homeostasis.^40^ Such modifications in OATP1B function have the potential to affect drug therapy and disposition, and we need to further elucidate the exact underlying mechanisms. In this study, we have demonstrated that OATP1B1 and OATP1B3 are *S*-palmitoylated on residue Cys24. Although this reversible PTM has historically impacted the individual function and surface expression of various transporters like apical sodium-dependent bile acid transporter, GLUT1, GLUT4, etc., our results demonstrate that OATP1B1 and OATP1B3 appear to deviate from this, with both individual surface expression and function not being affected after the loss of *S*-palmitoylation. Instead, our coexpression results suggest that *S*-palmitoylation regulates the protein-protein interactions of OATP1B1 and OATP1B3. The exact mechanism of how *S*-palmitoylation alters these protein-protein interactions is still being actively investigated. We currently postulate that *S*-palmitoylation at Cys24 causes a shift in OATP1B1 and OATP1B3 localization into cholesterol- and sphingolipid-enriched microdomains in the plasma membrane, traditionally called lipid rafts. If that is the case, then loss of *S*-palmitoylation would result in these transporters shifting out of the membrane microdomains, which could alter their proximity to one another and, in turn, impact their protein-protein interactions.

## Conflict of interest

The authors declare no conflicts of interest. Given his role as a member of the *Drug Metabolism and Disposition* Editorial Advisory Board, Bruno Hagenbuch had no involvement in the peer review of this article and had no access to information regarding its peer review.
