# Supplementary material for: Loss of palmitoylation impacts the interaction between the organic anion transporting polypeptide (OATP) 1B1 and OATP1B3
Source: Drug Metab Dispos. 2026 May 16;54(7):100330. doi: 10.1016/j.dmd.2026.100330 (PMC13494216; doi:10.1016/j.dmd.2026.100330)

Supplemental Figure 1. Comparison of substrate uptake mediated by tagged vs untagged OATP1B1 and OATP1B3. Uptake of 0.1 μM [^{3}H]-estradiol-17β-glucuronide (E17βG; 0.3 μCi/ml of radioactivity) (left side) by OATP1B1 transfected HEK293 cells or 0.1 μM [^{3}H]-cholecystokinin-8 (CCK-8; 0.3 μCi/ml of radioactivity) (right side) by OATP1B3 transfected HEK293 cells was measured for 20 seconds at 37 °C 48 hours after transfection. Results are shown as mean ± SD of a single experiment after subtracting values obtained with empty vector transfected cells.

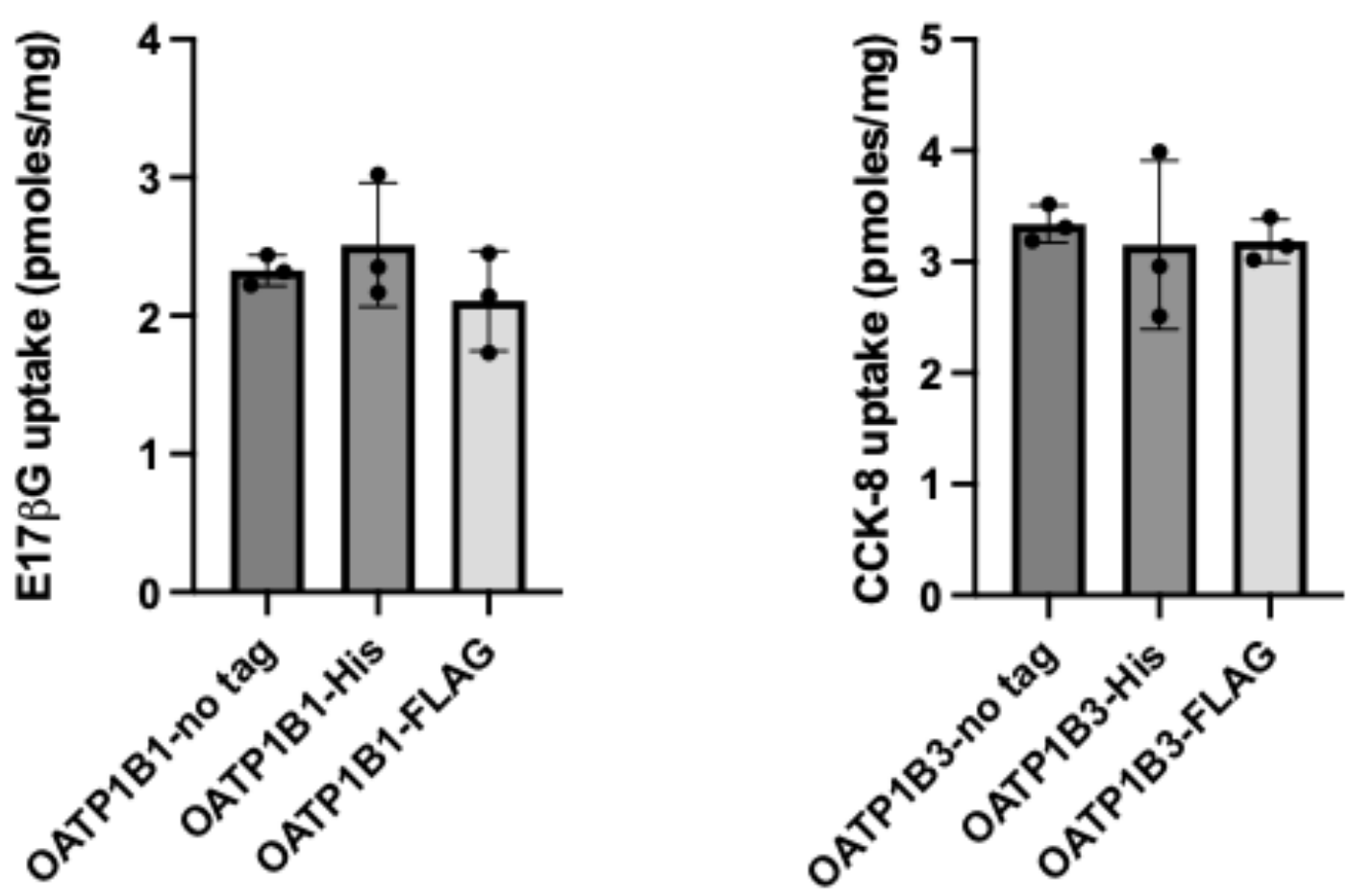

Supplemental Figure 2. Simplified Schematic of the Acyl-Resin Assisted Capture (RAC) assay. First, all free cysteine thiol groups in the target protein are blocked with a thiol blocking reagent (e.g., NEM) and selectively cleaved using hydroxylamine (HA+). The newly liberated thiol groups are then captured using thiopropyl sepharose resin and are affinity-purified with streptavidin. The captured proteins are then eluted and analyzed using western blotting. The image was created with BioRender.com.

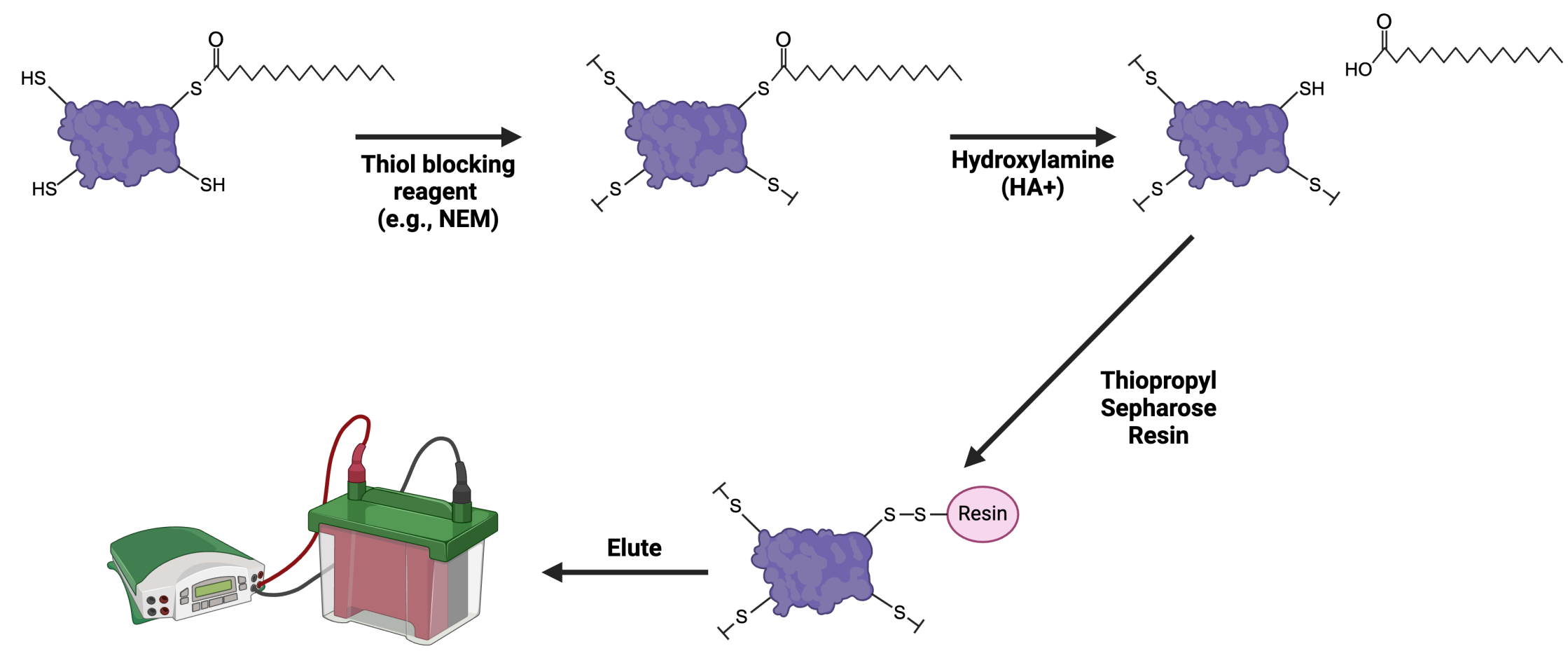

Supplemental Figure 3. Effect of 2-bromopalmitate (2BP) on OATP1B1-C24A and -C24S uptake function. The effect of 2BP on the function of non-palmitoylated OATP1B1 was assessed. Uptake of 0.1 µM [^{3}H]-estradiol-17β-glucuronide (E17βG; 0.3 µCi/ml of radioactivity) in OATP1B1 transiently transfected HEK293 cells was measured 48 hours after transfection for 20 seconds at 37 °C after 18 h treatment with increasing concentrations of 2BP. Both palmitoylated and non-palmitoylated OATP1B1 exhibited a dose-dependent decrease in function after treatment with 2BP, suggesting that the observed effect is not directly correlated with S-palmitoylation changes in OATP1B1.

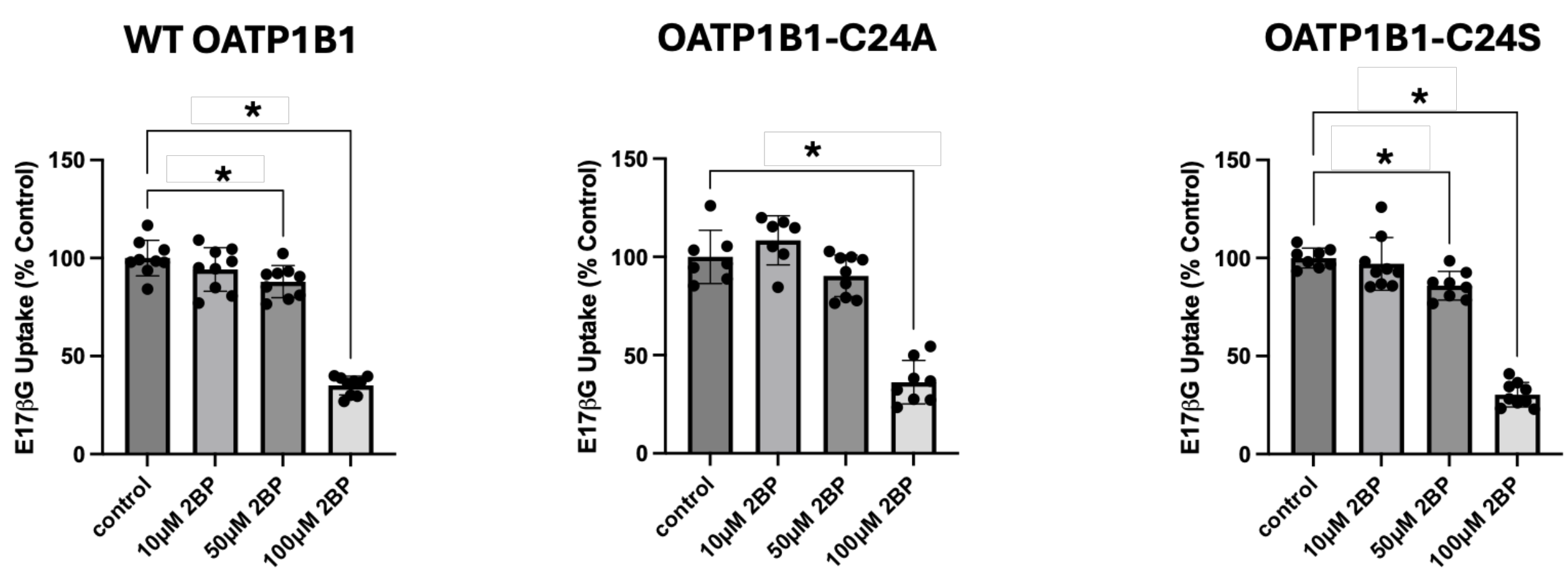

Supplement: Supplementary file 1 [file mmc1.pdf]
